# An evidence-based multi-factorial model to predict the oxygen cost of ventilation during ramp-incremental cycle ergometry exercise

**DOI:** 10.3389/fphys.2026.1702120

**Published:** 2026-02-19

**Authors:** Bridgette G. J. O’Malley, Robert A. Robergs, Karel Hrach, Chantal A. Vella, Derek W. Marks

**Affiliations:** 1 Faculty of Health: School of Exercise and Nutrition Sciences, Queensland University of Technology, Brisbane, QLD, Australia; 2 School of Health, Sport and Exercise Science, University of the Sunshine Coast, Brisbane, QLD, Australia; 3 Faculty of Health Studies, Jan Evangelista Purkyne University, Usti nad Labem, Czechia; 4 Department of Sports Medicine and Active Health Sciences, Faculty of Medicine, Charles University, Pilsen, Czechia; 5 Department of Movement Sciences, University of Idaho, Moscow, ID, United States; 6 Department of Kinesiology, Saint Mary’s College of California, Moraga, CA, United States

**Keywords:** oxygen cost of breathing, ventilation, ramp-incremental exercise, cycle ergometry, computational model

## Abstract

**Introduction:**

During maximal ramp-incremental exercise (RIE), the oxygen uptake–power output relationship ( $\dot{V}$ O_2_gain) may deviate from linearity near exhaustion. An increased oxygen cost of ventilation ( $\dot{V}$ O_2VENT_) is a plausible but under-quantified contributor. This study tested a non-linear multi-factorial model using measured $\dot{V}$ O_2VENT_ and six predictors: resting expired ventilation ( $\dot{V}$ _E_), weight, height, age, $\dot{V}$ O_2_ peak, and maximal heart rate (HRMax) to 1) estimate $\dot{V}$ O_2VENT_ and its contribution to maximal oxygen uptake ( $\dot{V}$ O_2_max) in an independent dataset and 2) determine whether correcting $\dot{V}$ O_2_ by $\dot{V}$ O_2VENT_ ( $\dot{V}$ O_2VCORR_) alters $\dot{V}$ O_2_max and $\dot{V}$ O_2_gain estimates.

**Methods:**

Published data from 42 participants (11 women, 31 men; 29 ± 6.5 years; $\dot{V}$ O_2_max = 4.02 ± 1.06 L min^−1^) were used to derive the model. Leave-one-out cross-validation (LOOCV) was used to assess validity, with predictive accuracy and coefficient stability evaluated via bootstrap resampling. The model was applied to an independent RIE dataset to generate $\dot{V}$ O_2VCORR_, which was compared with uncorrected $\dot{V}$ O_2_ across six %Wpeak intensities using repeated-measures ANOVA and final 30 s slope analysis.

**Results:**

The model explained 81% of $\dot{V}$ O_2VENT_ variance (adjusted *R* ^2^ = 0.78). $\dot{V}$ O_2VENT_ represented 17.43% ± 3.58% of $\dot{V}$ O_2_ at $\dot{V}$ O_2_max. Across 35%–100% Wpeak, $\dot{V}$ O_2VCORR_ values (L·min^−1^) increased with intensity (1.77 ± 0.43, 2.68 ± 0.57, 3.43 ± 0.72, 3.72 ± 0.79, 3.84 ± 0.86, and 3.92 ± 0.82) but remained significantly lower than uncorrected $\dot{V}$ O_2_ (*p* < 0.001), with the final-30 s $\dot{V}$ O_2_ slope attenuated following correction (p = 0.002).

**Conclusion:**

The internally validated model revealed $\dot{V}$ O_2VENT_ may contribute to a significant fraction of $\dot{V}$ O_2_ near exhaustion.

## Introduction

There has been growing interest in the presence of a non-linear profile of the oxygen uptake–power output relationship ( $\dot{V}$ O_2_gain) during maximal ramp-incremental exercise (RIE) (Iannetta et al., 2019; Hansen et al., 1988; Zoładź et al., 1995; Zoładź and Korzeniewski, 2001; Barstow et al., 2000; Scheuermann et al., 2002; Lucía et al., 2002a; Pedersen et al., 2002; Zoładź et al., 1998; Bickham et al., 2004; Lucía et al., 2002b; Boone and Bourgois, 2012). Original interpretations of this relationship described the $\dot{V}$ O_2_gain during RIE as constant, meaning that for RIE protocols applying a linear increase in exercise intensity (x-axis), the total oxygen uptake ( $\dot{V}$ O_2_) response (y-axis) to the protocol was also linear until, for some individuals, a plateau or deviation toward a plateau occurred at volitional exhaustion (Whipp et al., 1981; Davis et al., 1982).

Despite this historical context, it has been well-documented that significant variability exists between participants in the $\dot{V}$ O_2_gain of linear RIE protocols, with the $\dot{V}$ O_2_gain decreasing (Zoładź et al., 1998; Bickham et al., 2004), increasing (Hansen et al., 1988; Barstow et al., 2000; Scheuermann et al., 2002), displaying mixed non-linear responses (Lucía et al., 2002a; Pedersen et al., 2002; Zoładź et al., 1998), or remaining linear to volitional fatigue (Supplementary Figure S1). The increasing between-subject variability in this relationship and the incidence of a $\dot{V}$ O_2_ plateau have raised concerns about the validity of maximal oxygen uptake ( $\dot{V}$ O_2_max) determination and subsequent constant-work rate (CWR) exercise prescription (Iannetta et al., 2019; Niemeyer et al., 2021). Ultimately, gold-standard criteria for determining $\dot{V}$ O_2_max are yet to be established due to differences between researchers and laboratories in data processing strategies, the sensitivity and accuracy of different metabolic carts, large within-subject and between-subject variability in $\dot{V}$ O_2_gain profiles toward maximal exhaustion, the absence or presence of a $\dot{V}$ O_2_ plateau, and other physiological and data processing concerns (Boone and Bourgois, 2012).

Advancements in understanding $\dot{V}$ O_2_gain variability have revealed several possible physiological causes (Boone and Bourgois, 2012). Current insights suggest that increasing $\dot{V}$ O_2_gain may be the result of a combination of physiological factors, including increases in lactate, hydrogen ions, or catecholamines, muscle temperature, proton leaks through the inner mitochondrial membrane, decreases in cytosolic phosphorylation potential, increased cost of stabilizing muscles of the upper body, increases in the oxygen cost of ventilation ( $\dot{V}$ O_2VENT_), altered motor unit recruitment, and/or differences in fitness levels (Boone and Bourgois, 2012; Zoladz et al., 2002; Hug et al., 2004; Vella et al., 2006). The most widely explored, evidence-based physiological factors explaining variable $\dot{V}$ O_2_gain are muscle fiber type proportions (slow-twitch (ST) vs. fast-twitch (FT)) (Lucía et al., 2002a; Hug et al., 2004; Jones et al., 2004; Marles et al., 2006). A key limitation of prior interpretations is that the roles of other major contributors to $\dot{V}$ O_2_ cost, such as $\dot{V}$ O_2VENT,_ were not quantified or considered, leading to the prevailing interpretation that increasing $\dot{V}$ O_2_gain profiles result from greater recruitment of FT fibers (Lucía et al., 2002a; Zoladz et al., 2002; Hug et al., 2004; Jones et al., 2004; Marles et al., 2006). However, lower mitochondrial-derived adenosine triphosphate (ATP) turnover has been measured in FT fibers, which, hypothetically, should result in slow $\dot{V}$ O_2_ kinetics that reduce the ability to increase $\dot{V}$ O_2_, especially at the higher power outputs experienced at the end of an RIE test (Wakeling et al., 2006). Such responses would lower the $\dot{V}$ O_2_gain. Given these uncertainties and the insufficient research on FT fiber involvement, other contributors beyond skeletal muscle fiber type and percent distribution warrant further inquiry.

One such contributor is the $\dot{V}$ O_2VENT_, and the extent to which this measure increases above the gas-exchange threshold (GET) (Vella et al., 2006; Aaron et al., 1992a; Marks et al., 2005; Coast et al., 1993; Harms et al., 1997; Harms et al., 1998). The $\dot{V}$ O_2VENT_ is defined as the energy requirement of the elevated minute ventilation ( $\dot{V}$ _E_) by either exercise or voluntary hyperventilation. It is measured by completing multiple trials of breathing in seated (non-exercising) conditions, or other postures of interest, at $\dot{V}$ _E_ values similar to those at different exercise intensities. It is calculated as the difference in $\dot{V}$ O_2_ at rest and during hyperventilation (Vella et al., 2006; Marks et al., 2005). A greater understanding of $\dot{V}$ O_2VENT_ may provide an important avenue to deepen our understanding of $\dot{V}$ O_2_gain dynamics and allow for distinct quantification of its impact relative to skeletal muscle contributions to whole-body $\dot{V}$ O_2_ (wb $\dot{V}$ O_2_). For example, the disproportionate increase in $\dot{V}$ _E_, particularly at higher power outputs during an RIE test, has been proposed to explain increases in $\dot{V}$ O_2_gain not accounted for by muscle fiber proportions alone (Vella et al., 2006; Marks et al., 2005).

Bartlett et al. (1958) were the first to reveal an exponential increase in $\dot{V}$ O_2VENT_ during moderate to high rates of $\dot{V}$ _E_. The metabolic cost of this exponential increase was later quantified for untrained humans as being approximately 10% of $\dot{V}$ O_2_max (Shephard, 1966; Nielsen, 1936; Aaron et al., 1992b) and, in endurance-trained men, 15% of $\dot{V}$ O_2_max (Aaron et al., 1992a). Further research revealed the significant contribution of the $\dot{V}$ O_2VENT_ to wb $\dot{V}$ O_2_, with estimates between 5% and 18% of $\dot{V}$ O_2_max (Vella et al., 2006; Marks et al., 2005; Dominelli et al., 2014; Oueslati et al., 2017; Oueslati et al., 2016). Only two prior studies have quantified the impact of the $\dot{V}$ O_2VENT_ on calculations of $\dot{V}$ O_2_max between participants (Vella et al., 2006; Marks et al., 2005). Despite clear evidence that $\dot{V}$ O_2VENT_ constitutes a significant fraction of wb $\dot{V}$ O_2_ and to increasing $\dot{V}$ O_2_gain profiles (Vella et al., 2006; Marks et al., 2005), no standardized approach exists to systematically quantify this effect across different individuals and exercise intensities.

Direct measurement of $\dot{V}$ O_2VENT_ requires a higher percent carbon dioxide (CO_2_) gas to be breathed in during testing to prevent hypocapnia in higher $\dot{V}$ _E_ rate trials. This unique setup and access to medical-grade gas may be inaccessible in athletic testing environments. To address this gap, we developed a computational model based on previously published datasets to estimate $\dot{V}$ O_2VENT_ during RIE without requiring hyperventilation testing. An evidence-based model would provide a practical, scalable method for estimating $\dot{V}$ O_2VENT_ across individuals, enabling more accurate interpretation of $\dot{V}$ O_2_ and $\dot{V}$ O_2_gain during RIE and its underlying physiological components in both research and applied settings.

We aimed to test whether a non-linear multi-factorial model derived from measured $\dot{V}$ O_2VENT_ and six predictors: resting expired ventilation ( $\dot{V}$ _E_), weight, height, age, $\dot{V}$ O_2_ peak, and maximal heart rate (HRMax) could 1) estimate $\dot{V}$ O_2VENT_ and its contribution to maximal oxygen uptake ( $\dot{V}$ O_2_max) in an independent dataset and 2) determine whether correcting $\dot{V}$ O_2_ by the $\dot{V}$ O_2VENT_ ( $\dot{V}$ O_2VCORR_) alters $\dot{V}$ O_2_max and $\dot{V}$ O_2_gain estimates. We hypothesized that the model would accurately estimate $\dot{V}$ O_2VENT,_ representing approximately 15%–20% of $\dot{V}$ O_2_max, and that $\dot{V}$ O_2_ corrected by the $\dot{V}$ O_2VENT_ ( $\dot{V}$ O_2VCORR_) would be significantly lower than uncorrected $\dot{V}$ O_2_, resulting in a reduced $\dot{V}$ O_2_gain across increasing exercise intensities.

## Methods

### Data extraction and regression analysis

A multi-factorial non-linear regression model was developed to predict $\dot{V}$ O_2VENT_ using previously published $\dot{V}$ O_2VENT_ data from repeated-measures experimental research. However, the analyses and results presented in the manuscript are entirely novel and have not been published elsewhere. The published data (Table 1) were collected by Vella et al. (2006) from 20 healthy, non-smoking, recreational and/or endurance-trained men (n = 18) and women (n = 2) and by Marks et al. (2005) from 22 healthy, non-smoking men (n = 13) and women (n = 9). Permission was granted by the authors to reuse the published work to develop the model, which was also checked and approved against prior ethical guidelines, including the Declaration of Helsinki and the Common Rule. All $\dot{V}$ _E_ trial data of Vella et al. (2006) were first converted (Equation 1) from BTPS to STPD volume conditions to ensure similar volume conditions to the participants of Marks et al. (2005). All participant data from both studies were then exported and combined into a single MS Excel file.

$$\;\text{STPD}=\text{BTPS }*\;\left(273/\left(273+Btemp\right)\right)*\left(\text{PB }/\text{Atm}\right),$$

$$\text{STPD}=\text{BTPS }*\;\left(273/\left(273+37\right)\right)*\left(635/760\right),$$ (1)

where 273 is 0 °C expressed in Kelvin units (K); Btemp is body temperature ( °C); PB is the barometric pressure (mmHg), and Atm is the standard atmospheric pressure (mmHg).

**TABLE 1:** Individual participant data (n = 42) from Vella et al. (2006) and Marks et al. (2005) for multiple gas-exchange and anthropometric variables for maximal ramp-incremental exercise (RIE) testing and breath-mimicking trials.

Participant	Ventilation ( $\dot{V}$ _E_)	Age (years)	Weight (kg)	Height (cm)	$\dot{V}$ O_2_ peak (L∙min^−1^)	MaxHR (bpm)	$\dot{V}$ O_2VENT_ (L∙min^−1^)
1	71.66	28	50.80	168.70	2.1681	191	0.42
2	149.29	27	71.30	188.70	4.1311	176	0.70
3	76.35	21	66.50	167.60	3.6010	185	0.50
4	87.85	23	38.10	171.10	2.3931	185	0.46
5	106.09	37	65.00	180.90	4.3459	172	0.67
6	107.18	33	72.50	171.70	3.7722	179	0.46
7	166.25	21	94.60	188.30	5.0810	186	0.73
8	105.23	22	60.40	171.20	3.2254	188	0.59
9	90.53	33	59.10	167.80	3.9077	197	0.50
10	122.81	30	78.00	185.00	4.6613	185	0.76
11	115.19	39	81.60	180.10	4.4480	171	0.56
12	142.14	43	87.00	179.00	3.4269	158	0.74
13	150.77	30	69.50	179.40	4.4765	174	0.70
14	150.85	32	87.70	181.30	6.5328	184	1.05
15	57.71	28	50.80	168.70	2.1681	191	0.42
16	139.88	26	83.60	184.70	4.5972	184	0.92
17	117.56	24	84.00	184.40	4.5368	160	0.53
18	143.44	26	93.20	195.70	5.5892	180	0.85
19	150.85	30	92.80	185.40	4.8572	175	1.05
20	125.98	25	76.80	184.40	4.8084	165	1.11
21	159.89	28	70.00	180.00	5.03	170	1.06
22	173.99	36	67.30	177.50	4.47	180	1.30
23	141.71	20	59.00	165.00	3.98	174	1.05
24	153.98	32	72.00	176.00	4.72	182	1.17
25	110.82	41	68.00	165.00	2.90	180	0.79
26	150.29	29	84.40	170.00	3.89	190	1.08
27	82.18	22	50.00	159.00	2.22	182	0.59
28	179.26	41	90.00	179.00	3.46	177	1.28
29	139.61	25	74.00	165.00	4.39	171	0.99
30	105.84	27	59.10	175.00	3.45	184	0.68
31	168.44	29	77.00	183.00	4.52	179	1.31
32	153.06	44	69.00	176.00	3.40	172	1.06
33	172.22	29	77.00	185.00	4.91	177	1.28
34	85.17	22	52.00	152.00	2.06	180	0.71
35	90.24	22	56.00	160.00	3.09	188	0.65
36	162.61	26	83.00	173.00	4.11	186	1.15
37	129.74	29	76.60	177.00	4.88	192	1.13
38	90.64	25	47.00	152.00	1.82	189	0.55
39	119.70	24	80.00	185.00	3.92	171	0.81
40	90.77	22	69.00	170.00	3.95	183	0.77
41	189.87	23	74.00	186.00	5.46	186	1.57
42	155.00	40	80.00	180.00	5.32	191	1.25
**Mean**	**128.158**	**29**	**71.374**	**175.586**	**4.016**	**180**	**0.855**
**SD**	**33.4298**	**6.5**	**13.6858**	**9.8480**	**1.0590**	**8.6**	**0.2976**

$\dot{V}$ E, ventilation; $\dot{V}$ O_2_ peak, highest oxygen uptake in liters per minute; MaxHR, maximum heart rate in beats per minute; $\dot{V}$ O_2VENT_, oxygen cost of ventilation in liters per minute; SD, standard deviation.

### Model development and statistical procedures

**FIGURE 1:**
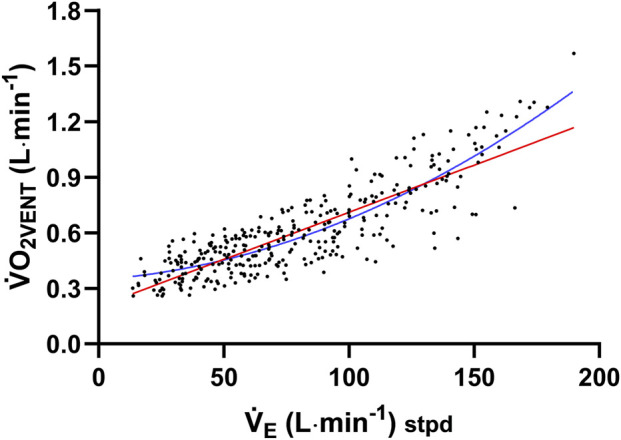
Total ventilation ( $\dot{V}$ _E_) data across multiple $\dot{V}$ _E_ mimicked trials against the oxygen cost of ventilation ( $\dot{V}$ O_2VENT_). Regression lines are shown for linear regression (red line) and a two-function polynomial (blue line). The respective equations are Y = 0.005091 × X + 0.2012 and Y = 0.3469 + 0.0009550 × X + 2.325e−005^2^.

All statistical analyses were performed using IBM SPSS Statistics v29.0.0.0, with results presented as mean ± standard deviation (SD) and significance set at *p* < 0.05. Model validation procedures, including leave-one-out cross-validation (LOOCV) and bootstrap resampling, were conducted in R (v4.3.1) using the caret and *boot* packages. Data normality was determined using Shapiro–Wilk tests, and potential outliers were identified by two authors through visual inspection of individual boxplots. Descriptive statistics summarized $\dot{V}$ _E_ and participant characteristics (Table 1). To determine the fit of $\dot{V}$ O_2VENT_ data for all participants (n = 42) of Vella et al. (2006) and Marks et al. (2005), the $\dot{V}$ _E_ rate across multiple $\dot{V}$ _E_ mimicked trials were plotted against the $\dot{V}$ O_2VENT_ (Figure 1). Linear and non-linear regression models were then compared to determine the best-fitting relationship, as shown in Figure 1.

**FIGURE 2:**
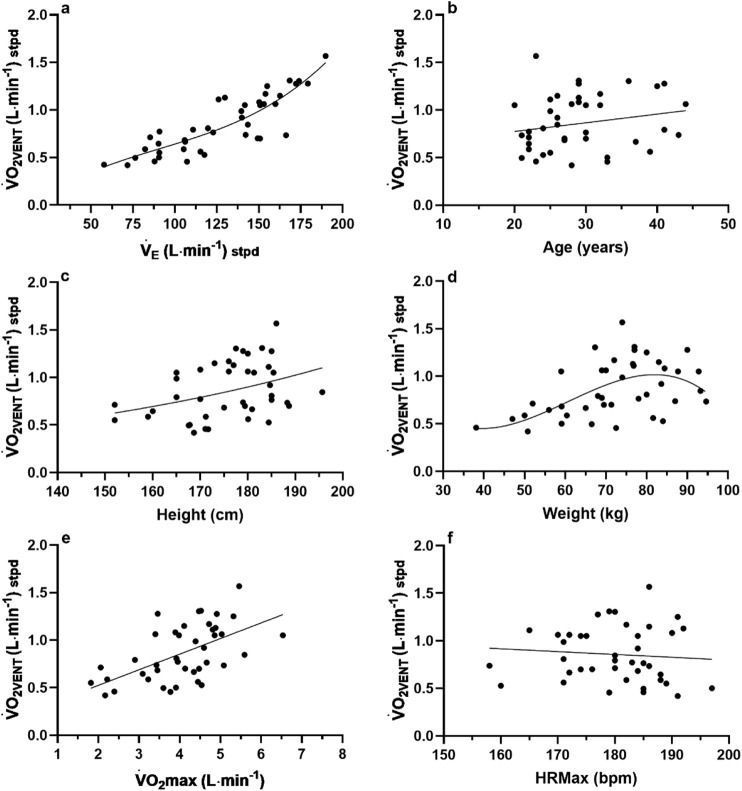
**(a–f)** Individual data (n = 42) for the subjects of Vella et al. (2006) and Marks et al. (2005) for the variables of interest for the $\dot{V}$ O_2VENT_ model.

To avoid collinearity due to repeated measures within the model dataset, only one $\dot{V}$ O_2VENT_ value per subject (peak $\dot{V}$ O_2VENT_) was used. All independent variables were assessed for multicollinearity using pairwise Pearson’s correlations (Supplementary Table S1). Variance inflation factors (VIF) for each predictor were obtained from a multiple regression model (Supplementary Table S2). The model was then developed based on the total individual participant data from Vella et al. (2006) and Marks et al. (2005) for the independent variables of $\dot{V}$ _E_, age (years), weight (kg), height (cm), $\dot{V}$ O_2_max (L∙min^−1^), maximum heart rate (HRMax; b∙min^−1^), and the $\dot{V}$ O_2VENT_ (L∙min^−1^) as presented in Figure 2. Each independent variable was plotted (GraphPad Prism, v10) against only the highest $\dot{V}$ O_2VENT_ (dependent variable) for each participant to adhere to the needed assumption of data independence for multiple regression analyses. Based on lowest standard error for each independent variable, the $\dot{V}$ _E_: $\dot{V}$ O_2VENT_ data (Figure 2a) were fitted with a non-linear third order polynomial (cubic), age: $\dot{V}$ O_2VENT_ data were fitted with a simple linear regression (Figure 2b); Height: $\dot{V}$ O_2VENT_ data were fitted with a non-linear exponential growth equation (Figure 2c); Weight: $\dot{V}$ O_2VENT_ data (Figure 2d) were fitted with a non-linear third order polynomial (cubic); $\dot{V}$ O_2_max: $\dot{V}$ O_2VENT_ data were fitted with a simple linear regression (Figure 2e); and HRMax: $\dot{V}$ O_2VENT_ data (Figure 2f) were fitted with a simple linear regression. The best-fit coefficient values of each independent variable and equation were then used to create the non-linear regression model. Please see “Full non-linear regression model expression.”

Equations 2–8 were developed for each independent variable based on the results of the linear or non-linear function applied to the participant data (Figures 2a–f).

$${\dot{V}}_{E}:\dot{V}O_{2VENT}$$

$$\begin{array}{ll} & V0+\left(V1\times VE\right)+\left(V2\times {VE}^{2}\right)+\left(V3\times {VE}^{3}\right))\\ & \;=-0.1918+\left(0.01515\times VE\right)+\left(-0.0001075\times {VE}^{2}\right)\\ & \;+\left(0.0000003927\times {VE}^{3}\right)), \end{array}$$ (2)

where V0, V1, V2, and V3 are the best-fit coefficient values, and VE is the highest $\dot{V}$ _E_ of the participant’s $\dot{V}$ _E_ trials.

$$Age:\dot{V}O_{2VENT}$$

$$\left(\left(SLA\times \left(Age\right)\right)+YIA\right)=\left(\left(0.009138\times \left(Age\right)\right)+0.5916\right),$$ (3)

where SLA is the slope of $\dot{V}$ O_2VENT_:age, YIA is the y-intercept of $\dot{V}$ O_2VENT_:age, and age is the participant’s age in years.

$$Height:\dot{V}O_{2VENT}$$

$$\left(\left(SLH\times \mathrm{EXP}\left(KH\times Height\right)\right)=\left(0.08766\times \mathrm{EXP}\left(0.01293\times Height\right)\right),\right.$$ (4)

where SLH is the slope of height, KH is the constant of height, and height is the height of the participant.

$$Weight:\dot{V}O_{2VENT}$$

$$\begin{array}{ll} & \left(WT0+\left(WT1\times Weight\right)+\left(WT2\times {Weight}^{2}\right)+\left(WT3\times {Weight}^{3}\right)\right)\\ & \;=\left(2.840+\left(-0.1442\times Weight\right)+\left(0.002706\times {Weight}^{2}\right)\right.\\ & \;\left.+\left(-0.00001486\times {Weight}^{3}\right)\right), \end{array}$$ (5)

where Wt0, Wt1, Wt2, and Wt3 are the best-fit coefficient values, and Weight is the weight of the participant in kg.

$$\dot{V}O_{2}\max:\dot{V}O_{2VENT}$$

$$\left(\left(VO2SL\times \left(VO2YI\right)\right)+PYI\right)=\left(\left(0.1643\times \left(VO2\max\right)\right)+0.1960\right),$$ (6)

where $\dot{V}$ O_2_SL is the slope of $\dot{V}$ O_2VENT_: $\dot{V}$ O_2_max, $\dot{V}$ O_2_YI is the y-intercept of $\dot{V}$ O_2VENT_: $\dot{V}$ O_2_max, and $\dot{V}$ O_2_max is the measured $\dot{V}$ O_2_max of the participant in L·min^−1^.

$$HRMax:\dot{V}O_{2VENT}$$

$$\left(\left(SLHR\times \left(HRMax\right)\right)+YIHR\right)=\left(\left(-0.002975\times \left(HRMax\right)\right)+1.392\right)$$ (7)

where SLHR is the slope of $\dot{V}$ O_2VENT_:HRMax, YIHR is the y-intercept of $\dot{V}$ O_2VENT_:HRMax, and HRMax is the maximum heart rate of the participant expressed as beats·min^−1^.

Full non−linear regression model expression

$$\begin{array}{ll} & {PREDVO}_{2\mathrm{VENT}}=57.398416273464+\left(-0.000970005711277\times \text{VE}\right)\\ & \;+\left(0.000017651237164\times {\text{VE}}^{2}\right)\\ & \;+\left(0.000000082921\times {\text{VE}}^{3}\right)\\ & \;+\left(5.6212655970623+\left(-0.110606151444052\times \text{Weight}\right)\right.\\ & \;+\left(0.001874750053414\times {\text{Weight}}^{2}\right)\\ & \;\left.+\left(-0.00001002\times {\text{Weight}}^{3}\right)\right)\\ & \;+\left(-144.186859758223\times \mathrm{EXP}\left(0.00006302\times \text{Height}\right)\right)\\ & \;+\left(\left(-0.00053642470208\times \left(\text{Age}\right)\right)+38.4177594334086\right)\\ & \;+\left(\left(0.062213494704466\times \left(\text{VO}2\text{peak}\right)\right)\right.\\ & \;\left.+21.2264109748025\right)\\ & \;+\left(\left(0.001880224895256\times \left(\text{HRMax}\right)\right)\right.\\ & \;\left.+25.042348428592.\right) \end{array}$$ (8)

The model’s ability to then estimate $\dot{V}$ O_2VENT_ was assessed by applying the model equation to previously published maximal RIE (ramp function Watts = 36 ± 3) data of 14 participants from the study of O’Malley et al. (2024). All gas-exchange data were measured breath-by-breath and, subsequently, averaged over seven breaths. The parameter estimates from the final iteration of the multi-factor non-linear regression model (Supplementary Table S3) were used to create a custom, functional computational model program in LabVIEW (National Instruments, Austin, TX, United States, v2017). This custom program was then used to predict the $\dot{V}$ O_2VENT_ of participants from the study of O’Malley et al. (2024) and to generate a graph of $\dot{V}$ O_2_max, $\dot{V}$ O_2VCORR_, and $\dot{V}$ O_2VENT_. The results of the predicted $\dot{V}$ O_2VENT_ of these participants are reported in Results.

### Statistical analysis procedures

#### Internal validation and predictive performance

Due to the modest sample size (n = 42), internal validation was performed for six predictors ( $\dot{V}$ _E_, Weight, Height, Age, $\dot{V}$ O_2_ peak, and HRMax) using LOOCV. LOOCV systematically holds out each observation in turn, fits the model to the remaining data, and predicts the outcome for the held-out observation. This procedure provides nearly unbiased estimates of predictive performance while mitigating overfitting. Model performance was quantified using adjusted *R* ^2^ to account for the number of predictors relative to the sample size, RMSE, and mean absolute error (MAE), calculated from LOOCV-predicted vs. observed values. Observed vs. predicted plots, as well as residuals vs. predicted plots, were generated to visually assess model fit and calibration. Shaded areas in calibration plots indicate the 95% confidence intervals for predicted values, illustrating uncertainty in individual predictions.

To further characterize uncertainty, bootstrap resampling of the LOOCV framework was conducted with 500 bootstrap iterations. For each iteration, the dataset was sampled with replacement, the model was refitted using the same LOOCV procedure, and both predicted values and model coefficients were recorded. This allowed calculation of 95% bootstrap confidence intervals for model performance metrics (*R* ^2^, RMSE, and MAE) and for each model coefficient to assess that the predictor’s contributions are consistent across resampled datasets.

#### Model predictions and calibration

Observed values represent measured $\dot{V}$ O_2VENT_, while predicted values are the corresponding LOOCV predictions. Observed vs. predicted values were plotted to assess calibration and predictive accuracy. Calibration analysis was performed by fitting a linear regression of predicted on observed values, with the slope and intercept reported to quantify systematic bias and prediction dispersion. The distribution of predicted $\dot{V}$ O_2VENT_ values across bootstrap iterations was summarized and visualized as histograms to illustrate the stability and variability of model performance.

The model was then applied to an independent sample to predict and compare the predicted $\dot{V}$ O_2VENT_ for participants from the study by O’Malley et al. (2024) across six different %Wpeak intensities via a one-way repeated-measures analysis of variance (ANOVA). A two-way ANOVA was performed to assess for any significant differences between $\dot{V}$ O_2_max and $\dot{V}$ O_2VCORR_ across these intensities, with follow-up pairwise comparisons for significant interactions. Finally, slope analyses of the final 30 s of $\dot{V}$ O_2_max and $\dot{V}$ O_2VCORR_ were determined using simple linear regression, with differences between slopes evaluated via paired samples t-tests. Descriptive analyses were performed for the 14 participants of O’Malley et al. (2024) for $\dot{V}$ O_2VENT and_ $\dot{V}$ O_2_max and the % $\dot{V}$ O_2VENT_ of $\dot{V}$ O_2_max was calculated as the difference between $\dot{V}$ O_2VENT and_ $\dot{V}$ O_2_max multiplied by 100.

## Results

### Model performance

The non-linear regression model converged after 588 major iterations, with the residual sum of squares decreasing significantly from 777.662 to 0.686, meeting the convergence criterion of a relative reduction in residual sum of squares at most 1.000 × 10^−8^. The estimated coefficients, standard error, and 95% confidence intervals for each constant are presented in Table 2. The model explained variance in $\dot{V}$ O_2VENT_, F (16, 26) = 19.983, *p* < 0.001, *R* ^2^ value of 0.81, indicating that 81.1% of the variance in $\dot{V}$ O_2VENT_ was accounted for by the independent variables. Although moderate correlations were observed between some anthropometric and physiological variables (e.g., $\dot{V}$ O_2_max and height, r = 0.761), all pairwise correlations (Supplementary Table S1) were below the commonly accepted multicollinearity threshold (r = 0.80). VIFs for all independent variables were also <5 (Supplementary Table S2).

**TABLE 2:** Individual participant data (n = 14) for gas-exchange and ventilatory variables.

Participant	Sex	$\dot{V}$ O_2_max (L∙min^−1^)	$\dot{V}$ O_2VENT_ (L∙min^−1^)	$\dot{V}$ O_2VENT_% of $\dot{V}$ O_2_max
1	M	5.90	0.91	15.42
2	M	5.69	0.75	13.18
3	M	5.09	0.93	18.27
4	M	5.41	0.77	14.23
5	F	3.80	0.61	16.05
6	M	5.91	1.1	18.61
7	M	4.97	0.88	17.71
8	F	2.24	0.53	23.66
9	M	4.94	1.1	22.27
10	M	4.24	0.59	13.92
11	M	4.42	0.92	20.81
12	M	4.87	0.95	19.51
13	M	4.33	0.8	18.48
14	M	4.85	0.58	11.96
**Mean**		**4.761**	**0.816**	**17.434**
**SD**		**0.9591**	**0.1868**	**3.4835**

$\dot{V}$ O_2_max, maximal oxygen uptake; $\dot{V}$ O_2VENT_, oxygen cost of ventilation; SD, standard deviation.

**FIGURE 3:**
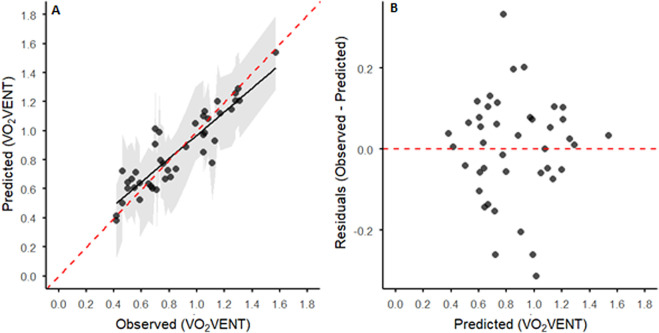
**(A)** Predicted vs. observed $\dot{V}$ O_2VENT_ values (n = 42) for all participants based on leave-one-out cross-validation (LOOCV) predictions. The black line shows the linear regression of predicted values on observed values (calibration line). The dashed red line represents the ideal 1:1 parity line, with the surrounding gray-shaded areas displaying the prediction interval (PI) for individual predicted values based on the LOOCV results. **(B)** Residuals vs. predicted $\dot{V}$ O_2VENT_ values for the LOOCV model. Residuals were calculated as the difference between observed and predicted $\dot{V}$ O_2VENT_. The horizontal dashed red line indicates zero residuals.

LOOCV indicated good model performance, with RMSE = 0.128, MAE = 0.098, *R* ^2^ = 0.812, and adjusted *R* ^2^ = 0.78, accounting for the six predictors relative to the sample size. Observed vs. predicted $\dot{V}$ O_2VENT_ values showed good alignment (calibration slope = 0.81, intercept = 0.16), and residuals vs. predicted plots indicated no systematic deviation across the prediction range (Figures 3A,B).

Bootstrap resampling of the LOOCV predictions (500 iterations) generated 95% confidence intervals for model metrics: [RMSE = 0.128, 95% CI (0.098, 0.155); MAE = 0.098, 95% CI (0.074, 0.124); *R* ^2^ = 0.78, 95% CI (0.68, 0.90)]. Histograms of the bootstrap distributions (Supplementary Figure S2) illustrate the variability in predictive performance across resamples. Bootstrap-derived 95% confidence intervals for model coefficients are summarized in Supplementary Table S4. Some intervals included zero (e.g., Intercept: −5.36 to 15.17; $\dot{V}$ _E_ linear term: −0.0616 to 0.0734), reflecting expected variability given the sample size and number of predictors. However, the model shows moderate predictive accuracy and calibration despite these uncertainties.

### Application of the model to an independent dataset

**FIGURE 4:**
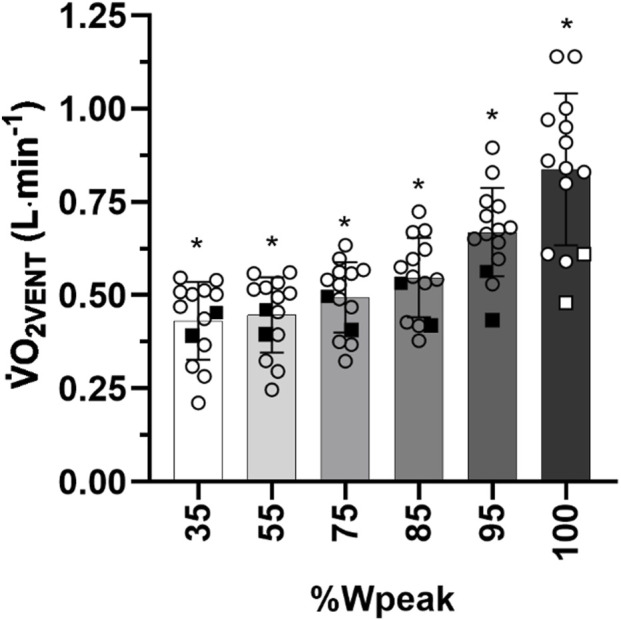
Mean ± SD of maximal oxygen uptake ( $\dot{V}$ O_2_max; L∙min^−1^) and $\dot{V}$ O_2_ corrected by the oxygen cost of breathing ( $\dot{V}$ O_2VCORR_) at 35%, 55%, 75%, 85%, 95%, and 100% of maximal power output (Wpeak) for 14 highly trained subjects of O’Malley et al. (2024). *Significant difference (*p* < 0.05). Filled squares and open squares = two women.

Results of the one-way repeated-measures ANOVA with the Greenhouse–Geisser correction revealed a significant main effect for predicted $\dot{V}$ O_2VENT_ across the different intensity points, *F* (1.129, 14.681) = 37.680, *p* < 0.001 (Figure 4).

Individual participant data are presented in Table 2 for $\dot{V}$ O_2VENT,_ $\dot{V}$ O_2_max, and the % $\dot{V}$ O_2VENT_ of $\dot{V}$ O_2_max. At $\dot{V}$ O_2_max, $\dot{V}$ O_2VENT_ expressed as a percentage of $\dot{V}$ O_2_max, was 17.43% ± 3.48% (Table 2).

**FIGURE 5:**
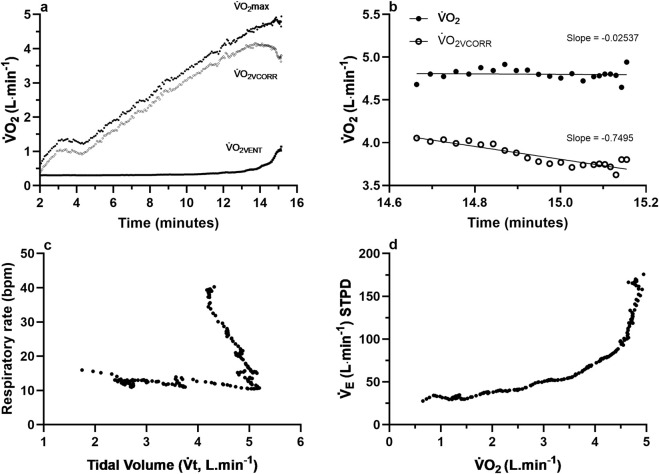
**(a)** Raw demographic data of one representative subject from O’Malley et al. (2024) for measures of maximal oxygen uptake ( $\dot{V}$ O_2_max), oxygen uptake ( $\dot{V}$ O_2_) corrected by the oxygen cost of ventilation ( $\dot{V}$ O_2VCORR_), and the predicted oxygen cost of ventilation ( $\dot{V}$ O_2VENT_); **(b)** the slope of $\dot{V}$ O_2_ and $\dot{V}$ O_2VCORR_ for the final 30 s of the ramp-incremental exercise (RIE) test for the same participant; **(c)** the respiratory rate (breaths per minute) against tidal volume (Vt; L∙min^−1^) for the duration of the RIE test; **(d)** expired ventilation ( $\dot{V}$ _E;_ L∙min^−1^) against measured $\dot{V}$ O_2_ for the duration of the RIE test.

Raw data of one participant are presented in Figures 5a–d for $\dot{V}$ O_2_max, $\dot{V}$ O_2VCORR_, and $\dot{V}$ O_2VENT._ The mean slope (final 30 s) of $\dot{V}$ O_2_max was significantly greater (0.2866 ± 0.2549 L min^−1^) than the mean slope of $\dot{V}$ O_2VCORR_, (0.0419 ± 0.3954 L min^−1^; *t* (13) = 4.003; p = 0.002, *d* = 1.07).

**FIGURE 6:**
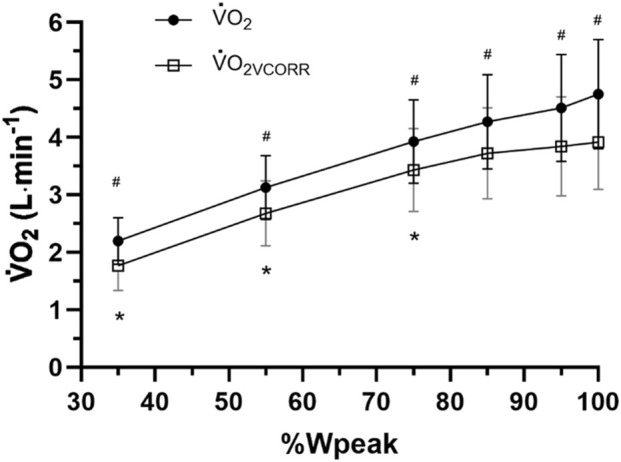
Mean ± SD uncorrected oxygen uptake ( $\dot{V}$ O_2_) vs. $\dot{V}$ O_2_ corrected ( $\dot{V}$ O_2VCORR_) for the oxygen cost of breathing ( $\dot{V}$ O_2VENT_) for 14 highly trained cyclists of O’Malley et al. (2024) at 35%, 55%, 75%, 85%, 95%, and 100% of maximal power output (Wpeak). *Significant difference (*p* < 0.05) across intensities.

Two-way repeated-measures ANOVA with Greenhouse–Geisser correction revealed a significant main effect for intensity, F (1.31, 17.02) = 115.66, *p* < 0.001; measure, F (1, 13) = 533.05, *p* < 0.001; and a significant two-way interaction between intensity and measure, F (1.13, 14.67) = 37.46, p = <0.001 (Figure 6). Follow-up pairwise comparisons revealed a significant difference between $\dot{V}$ O_2_ and $\dot{V}$ O_2VCORR_ across all levels of intensity (*p* < 0.001; Figure 6). Furthermore, $\dot{V}$ O_2_ was significantly different between all levels of intensity (*p* < 0.001; Figure 6) but $\dot{V}$ O_2VCORR_ was only significantly different across 35%, 55%, and 75% Wpeak (*p* < 0.001) but not between 85% and 95% (p = 0.927); 85% and 100% (p = 0.269); and for 95% and 100% (*p* = 1.00; Figure 6).

## Discussion

The present study set out to develop and apply an evidence-based computational model for estimating $\dot{V}$ O_2VENT_ without requiring additional hyperventilation-mimicking trials, and the findings are threefold. First, the model demonstrated close agreement between predicted and measured $\dot{V}$ O_2VENT_ values (Figures 3A,B), reflecting good calibration and stable performance across participants. Second, when the model was applied to a new dataset of trained participants, the mean predicted $\dot{V}$ O_2VENT_, expressed as a percentage of $\dot{V}$ O_2_max, was 17.43% ± 3.48% (Figure 4; Table 2; range 11.96%–23.66%), in line with several previous findings. Finally, the mean slope (final 30 s) of uncorrected $\dot{V}$ O_2_ was significantly greater (0.2866 ± 0.2549 L min^−1^) than that of $\dot{V}$ O_2VCORR_ (Figures 5, 6), highlighting the potentially significant contribution of $\dot{V}$ O_2VENT_ to total $\dot{V}$ O_2_ and, consequently, to the increase in $\dot{V}$ O_2_ gain near maximal exhaustion during RIE.

### Model performance and $\dot{V}$ O_2VENT_ contribution to $\dot{V}$ O_2_max

The developed model demonstrated good apparent predictive performance and reasonable calibration for estimating $\dot{V}$ O_2VENT_ (Figures 3A,B; Supplementary Figure S2; Supplementary Table S4). While bootstrap resampling of the LOOCV predictions (500 iterations) indicated moderate variability in performance estimates, reflecting some sample sensitivity inherent to small datasets, the general pattern of predictive behavior remained consistent (Supplementary Figure S2; Supplementary Table S4). Importantly, when the model was applied to an independent dataset, the predicted $\dot{V}$ O_2VENT_ values aligned closely with previously published observations, supporting its practical applicability and internal validity. These findings suggest that, despite expected uncertainty, the model provides a useful exploratory framework for estimating $\dot{V}$ O_2VENT_ and warrants further evaluation in larger and more diverse samples to confirm generalizability.

When applied to a separate dataset of trained cyclists, the predicted average contribution of $\dot{V}$ O_2VENT_ to $\dot{V}$ O_2_max was 17.43% ± 3.58%, with individual values ranging from 12% to 24% (Figure 4). These estimates closely align with previous observations of the contribution of $\dot{V}$ O_2VENT_ to $\dot{V}$ O_2_max of approximately 10%–18% in trained populations (Aaron et al., 1992a; Harms et al., 1997).

While the model has not yet undergone full external cross-validation with independent participant data, the LOOCV and bootstrap results, along with the agreement with existing datasets, suggest generally consistent predictive behavior (see Limitations). Importantly, this model is intended as an exploratory framework that opens a new avenue for investigating ventilatory function during exercise. At the same time, the relatively wide bootstrap confidence intervals for some coefficients, the small sample size, and the risk of overfitting suggest caution when applying the model to other datasets, underscoring the value of further validation in larger, independent samples. The observed inter-individual variability, including sex-based differences in $\dot{V}$ O_2VENT_ and $\dot{V}$ _E_, demonstrates the potential of this exploratory framework to enhance the interpretation of complex physiological responses, once further validation is performed (Table 2). Although the predicted $\dot{V}$ O_2VENT_ was lower in women than in men (Table 2), $\dot{V}$ O_2VENT_ as a percentage of $\dot{V}$ O_2_max was higher, suggesting that women may need to expend a greater percentage of their overall $\dot{V}$ O_2_ on breathing to achieve adequate $\dot{V}$ _E_. Prior literature (Espinosa-Ramírez et al., 2021; Sheel and Guenette, 2008) also supports men having larger overall metabolic rates, ventilatory capacities, and increased ventilatory efficiency, which is further corroborated by the lower relative, predicted $\dot{V}$ O_2VENT_ as a percentage of $\dot{V}$ O_2_max in the present study. Similar physiological and anatomical differences have also been observed by Dominelli et al. (2014), with higher relative ventilatory values of 13.8% and 9.4% of wb $\dot{V}$ O_2_ for women and men, respectively. The model’s consistency in producing $\dot{V}$ O_2VENT_ estimates aligned with prior observations without requiring additional hyperventilation protocols may support its biological plausibility, at least in trained populations.

The inter-individual variability of predicted $\dot{V}$ O_2VENT_ is not surprising, as the dataset of O’Malley et al. (2024) consisted of average $\dot{V}$ O_2_max values of 4.76 L min^−1^, with individual values ranging from 2.24 L min^−1^ to 5.91 L min^−1^ (Figure 4; Table 2). There is sufficient evidence to support physiological and anatomical differences between sexes that may explain the increasingly higher measured and, in the present study, estimated $\dot{V}$ O_2VENT_ for men compared to women (Dominelli et al., 2014; Espinosa-Ramírez et al., 2021). The lowest predicted $\dot{V}$ O_2VENT_ values at $\dot{V}$ O_2_max in the present study was 0.53 L min^−1^ and 0.61 L min^−1^, which, relative to $\dot{V}$ O_2_max, represented 23.66% and 16.05%, respectively (Figure 4; Table 2). Both observations involved two female participants from the O’Malley et al. dataset (O’Malley et al., 2024). Interestingly, the 23.66% value for $\dot{V}$ O_2VENT_ as a percent of $\dot{V}$ O_2_max was for a female participant who had the lowest absolute $\dot{V}$ O_2_max (participant 8; Table 2) in the present study. Some of the lowest predicted $\dot{V}$ O_2VENT_ values relative to $\dot{V}$ O_2_max were observed for men who had larger absolute $\dot{V}$ O_2_max values (Table 2). During dynamic exercise, women may experience increased resistive respiratory and elastic work, lower forced vital capacity, and high limited expiratory flow, which may explain the predicted increase in their $\dot{V}$ O_2VENT_ compared to their male counterparts (Sheel and Guenette, 2008).

### Non-linear $\dot{V}$ O_2_–PO dynamics

During continuous RIE to volitional exhaustion, some participants may demonstrate an increased $\dot{V}$ O_2_gain simultaneous with a non-linear increase in $\dot{V}$ O_2VENT_ above the GET (Vella et al., 2006; Marks et al., 2005). A similar $\dot{V}$ O_2_–PO dynamic was observed in the present study (Figures 5a–d), where the mean slope (final 30 s) of $\dot{V}$ O_2_ was significantly greater (0.2866 ± 0.2549 L min^−1^; p = 0.002; Figures 5a,b) than $\dot{V}$ O_2VCORR_ (0.0419 ± 0.3954 L min^−1^; Figure 5b), highlighting the significant contribution of $\dot{V}$ O_2VENT_ near volitional exhaustion. This is further supported by the disproportionate increase in predicted $\dot{V}$ O_2VENT_ with increasing PO intensities (35%–100% Wpeak; Figure 4), consistent with previous estimates attributing up to 18% of total $\dot{V}$ O_2_ during maximal RIE to ventilatory cost (Vella et al., 2006; Harms et al., 2000; Dominelli et al., 2014). The phenomenon reflects the known non-linear increase in ventilatory drive and increased work of breathing (WOB) near volitional exhaustion (Vella et al., 2006; Harms et al., 1997; Dominelli et al., 2014).

While predicted $\dot{V}$ O_2VENT_ increased simultaneously with increasing PO across the RIE test (Figure 6), the slope of $\dot{V}$ O_2VCORR_ was substantially attenuated, with no significant differences observed between 85%, 95%, and 100% Wpeak (Figure 6). This plateau in $\dot{V}$ O_2VCORR_, despite continued increases in total $\dot{V}$ O_2_ with increasing PO, suggests that increased $\dot{V}$ O_2_gain is more likely to be the result of increasing $\dot{V}$ O_2VENT_ rather than by further increases in locomotor muscle oxygen uptake due to increased motor unit (predominantly FT) recruitment.

The increasing $\dot{V}$ O_2VENT_ observed toward volitional exhaustion (Figures 4, 5a) is consistent with the known ventilatory response during high-intensity exercise, wherein $\dot{V}$ _E_ increases exponentially, primarily through elevations in breathing frequency (fR) once tidal volume (Vt) plateaus (Vella et al., 2006; Aaron et al., 1992a). However, individual responses in the present study varied. Some participants maintained or further increased Vt alongside fR, whereas others exhibited a leveling response in Vt at intensities above the GET, and others showed a decrease in Vt during the last minutes of the RIE. For these latter individuals, minute ventilation ( $\dot{V}$ _I_) continued to increase due to a rapidly increasing fR (Figures 5c,d). This shift could be hypothesized to reflect mechanical or neuromuscular limitations on tidal expansion, including dynamic hyperinflation, elevated intrinsic positive end-expiratory pressure, or respiratory muscle fatigue. These factors together may explain the increase in fR rather than depth and therefore, the less efficient $\dot{V}$ _E_ patterns and higher $\dot{V}$ O_2VENT_ observed (Harms et al., 1998; Dempsey et al., 2006). Limits to further increases in Vt and the need to increase fR reflect mechanical limitations to $\dot{V}$ _I_ that result in an increased WOB, particularly in smaller or less trained individuals, where the increased fR causes larger increases in $\dot{V}$ O_2VENT_ for given increments in $\dot{V}$ _E_. In other words, the increased ventilatory demand is met less efficiently, with a higher fR driving a disproportionate increase in $\dot{V}$ O_2VENT_. This is further exacerbated in individuals who have a declining Vt near the end of the RIE.

While earlier research has often emphasized increased FT fiber recruitment as the likely explanation for increased $\dot{V}$ O_2_gain near and/or at volitional exhaustion (Nielsen, 1936), our findings suggest that ventilatory mechanics and their oxygen cost must at least be considered in parallel. This is especially important for subjects with less efficient breathing strategies, for whom a high fR and reduced Vt may increase WOB and elevate $\dot{V}$ O_2VENT_ disproportionately.

### Perspective

The novel, non-linear regression model developed in this study provides a new method for estimating $\dot{V}$ O_2VENT_ using commonly derived RIE measures. Such modeling may provide a new exploratory avenue for examining physiological sex-based differences in $\dot{V}$ _E_ mechanics, which is an area of research in which female participants remain markedly underrepresented across age, health status, and fitness level. Future research should also aim to develop similar models within clinical contexts, and evaluation of this model’s applicability to independent datasets may continue to pave the way for greater reliance on modeling approaches to estimate difficult-to-measure physiological variables in performance testing and physiological data interpretation.

### Limitations

Limitations of the present study may include the risk of overfitting despite acceptable LOOCV performance, as the model was developed and validated using the same dataset. External validation using a new dataset of measured $\dot{V}$ O_2VENT_ may add to the robustness and generalizability of the model for future applications. Bootstrap confidence intervals for some model coefficients, reported in the Supplementary Material, were relatively wide and occasionally included zero. Similarly, some standard errors were larger than anticipated, highlighting uncertainty in specific coefficient estimates. Nevertheless, as this is the first study to model $\dot{V}$ O_2VENT_, the results infer high potential for further research inquiry on this topic. Additionally, because the model was developed based on the characteristics of healthy and trained individuals, it may only be applicable to a similar subset of participants. The findings of this study are specific to RIE protocols and may not directly generalize to step-incremental or CWR exercise. Finally, due to the inability to quantify $\dot{V}$ O_2VENT_ for resting $\dot{V}$ O_2_, the model assumes a similar resting $\dot{V}$ O_2_ to that measured by Vella et al. (2006) and Marks et al. (2005), even though it is logical that the $\dot{V}$ O_2VENT_ at rest must be a low-to-moderate percentage of the total resting $\dot{V}$ O_2_ measure.

## Conclusion

This study developed and internally validated a non-linear regression model to estimate the $\dot{V}$ O_2VENT_ during RIE without requiring hyperventilation-mimicking trials. The model showed strong agreement with the previously measured $\dot{V}$ O_2VENT_ data, and when applied to an established dataset, provided estimations of $\dot{V}$ O_2VENT_ that closely matched previous observations. Further, the model suggests that $\dot{V}$ O_2VENT_ may contribute to a significant and variable fraction of $\dot{V}$ O_2_ near and/or at volitional exhaustion that causes major adjustments to total $\dot{V}$ O_2_gain of the RIE in the last minutes of the protocol. The findings, although aligning with previous research, also reveal important individual and sex-based differences in ventilatory patterns during RIE. Notably, increases in $\dot{V}$ O_2_ near and/or at volitional exhaustion may be influenced by ventilatory demands, not only by increases in locomotor $\dot{V}$ O_2_. By providing a practical, evidence-based method for estimating $\dot{V}$ O_2VENT_, this work further reveals the capabilities to adjust whole-body $\dot{V}$ O_2_ data for a more valid limb skeletal muscle $\dot{V}$ O_2_ response. This has the potential to provide greater understanding of the physiological contributions of limb skeletal muscle to exercise-induced gas exchange, as well as improve the development, detection, and application of criteria used to verify the attainment of $\dot{V}$ O_2_max. As such, the results of this study suggest further research is needed to address a significant methodological gap in understanding the changing $\dot{V}$ O_2_gain of RIE, and how this could impact the detection of $\dot{V}$ O_2_max.

## Data Availability

The original contributions presented in the study are included in the article/Supplementary Material; further inquiries can be directed to the corresponding author.
