# Supplementary material for: An evidence-based multi-factorial model to predict the oxygen cost of ventilation during ramp-incremental cycle ergometry exercise
Source: Front Physiol. 2026 Feb 19;17:1702120. doi: 10.3389/fphys.2026.1702120 (PMC12960085; doi:10.3389/fphys.2026.1702120)
Supplement: Supplementary file 1 [file Supplementaryfile1.docx]

**Supplementary Material**


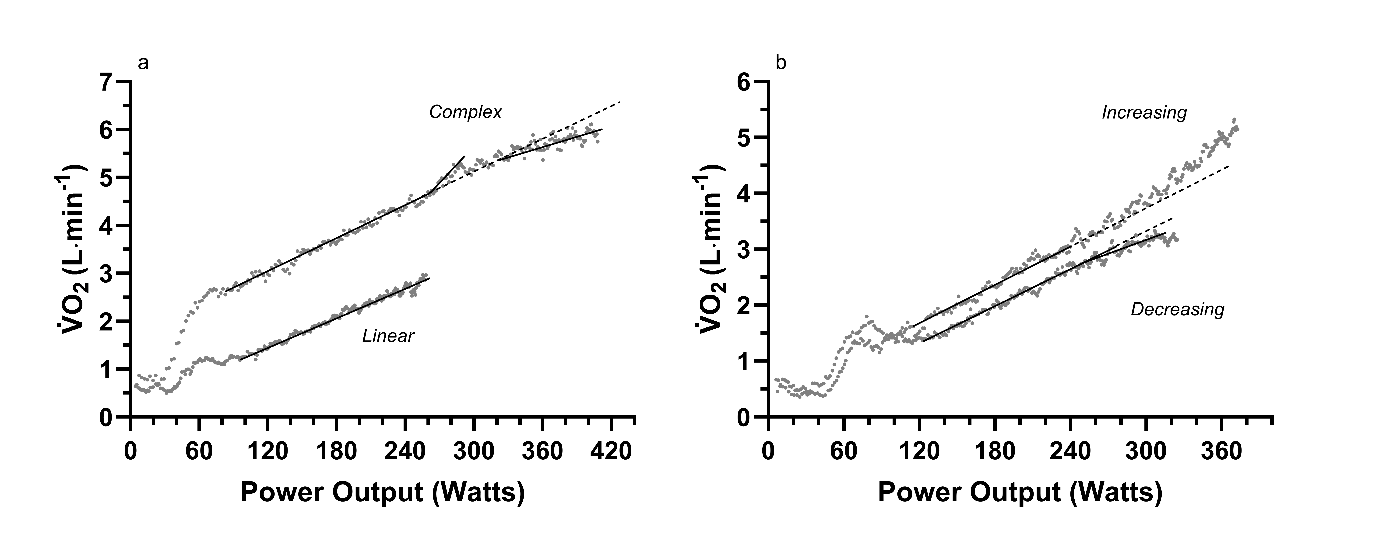


**Figure S1:** Representative data of four participants that reveal a) linear and complex V̇O_2_ responses to ramp-incremental exercise (RIE) and, b) examples of increasing and decreasing oxygen uptake (V̇O_2_)-power output (PO) relationships.


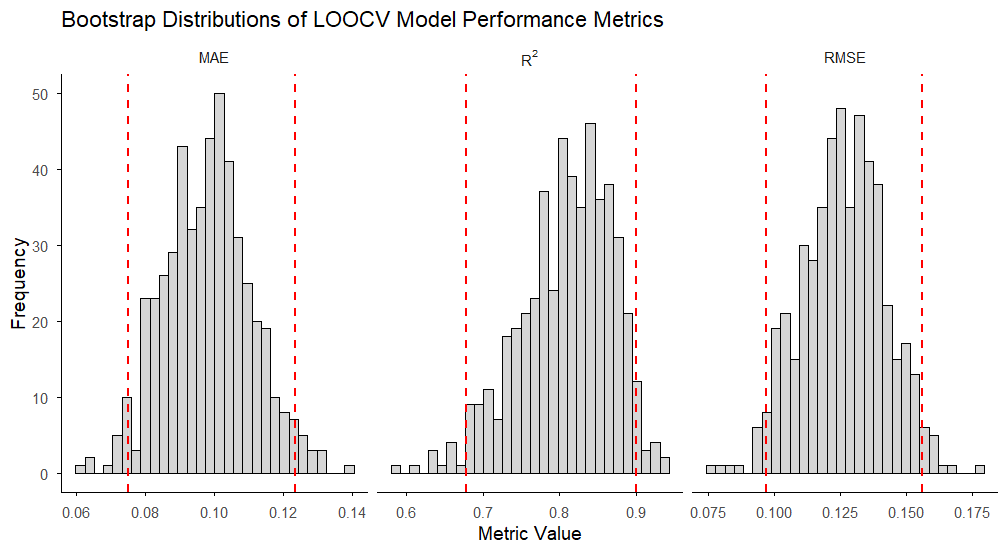


**Figure S2:** Bootstrap distributions of leave-one-out cross-validation (LOOCV) predictions metrics for V̇O₂_VENT_ predictions. The dataset was resampled with replacement 500 times, and the model was refitted on each bootstrap sample. Histograms show the distributions of (A) mean absolute error (MAE), (B) root mean squared error (RMSE), and (C) coefficient of determination (R²). Gray bars represent the frequency of bootstrap values for each metric. Vertical red dashed lines indicate the 95% confidence interval of the bootstrap distributions. Facets have independent X-axis scales to accommodate the different ranges of each metric.

**Table S1:**

| **Correlations** | | | | | | | | |
| --- | --- | --- | --- | --- | --- | --- | --- | --- |
|  |  | **VE** | **AGE** | **WEIGHT** | **HEIGHT** | **V̇O_2_P** | **MAXHR** |  |
| **VE** | Pearson Correlation | 1 | .282 | .687** | .613** | .698** | -.265 |  |
|  | Sig. (2-tailed) |  | .071 | <.001 | <.001 | <.001 | .089 |  |
| **AGE** | Pearson Correlation | .282 | 1 | .283 | .193 | .109 | -.217 |  |
|  | Sig. (2-tailed) | .071 |  | .069 | .222 | .493 | .167 |  |
| **WEIGHT** | Pearson Correlation | .687** | .283 | 1 | .727** | .749** | -.332* |  |
|  | Sig. (2-tailed) | <.001 | .069 |  | <.001 | <.001 | .032 |  |
| **HEIGHT** | Pearson Correlation | .613** | .193 | .727** | 1 | .761** | -.326* |  |
|  | Sig. (2-tailed) | <.001 | .222 | <.001 |  | <.001 | .035 |  |
| **V̇O_2_P** | Pearson Correlation | .698** | .109 | .749** | .761** | 1 | -.182 |  |
|  | Sig. (2-tailed) | <.001 | .493 | <.001 | <.001 |  | .249 |  |
| **MAXHR** | Pearson Correlation | -.265 | -.217 | -.332* | -.326* | -.182 | 1 |  |
|  | Sig. (2-tailed) | .089 | .167 | .032 | .035 | .249 |  |  |

^** Correlation is significant at the 0.01 level (2-tailed), * Correlation is significant at the 0.05 level (2-tailed). All independent variables have n=42 observations.^

**Table S2:**

| **Coefficients** | | | | | | | | |
| --- | --- | --- | --- | --- | --- | --- | --- | --- |
|  |  |  |  |  |  |  | **Collinearity Statistics** | |
| **Model** | | **Unstandardised B** | **Coefficients**  **Std. Error** | **Standardised**  **Coefficients**  **Beta** | **t** | **Sig.** | **Tolerance** | **VIF** |
| **(Constant)** | | .618 | .917 |  | .674 | .505 |  |  |
| **VE** | | .008 | .001 | .951 | 7.757 | <.001 | .429 | 2.331 |
| **AGE** | | .001 | .004 | .013 | .148 | .883 | .848 | 1.180 |
| **WEIGHT** | | -.003 | .003 | -.124 | -.874 | .388 | .322 | 3.105 |
| **HEIGHT** | | -.008 | .004 | -.272 | -1.996 | .054 | .348 | 2.872 |
| **V̇O_2_P** | | .065 | .043 | .233 | 1.531 | .135 | .279 | 3.589 |
| **MAXHR** | | .003 | .003 | .082 | .924 | .362 | .827 | 1.209 |

**Table footnote: Dependent Variable: V̇O_2VENT_**

**Table S3:** The final iteration and resulting parameter estimates for each constant included within the multi-factorial, non-linear regression model.

| **Parameter Estimates** | | | | |
| --- | --- | --- | --- | --- |
| **Parameter** | **Estimate** | **Std. Error** | **95% Confidence Interval** | |
|  |  |  | **Lower Bound** | **Upper Bound** |
| **B0** | 57.398 | .000 | 57.398 | 57.399 |
| **B1** | -.001 | .036 | -.074 | .072 |
| **B2** | 1.765e-5 | .113 | -.232 | .232 |
| **B3** | 8.292e-8 | .066 | -.135 | .135 |
| **WTB0** | 5.621 | .005 | 5.612 | 5.631 |
| **WTB1** | -.111 | 3419873.145 | -7029650.037 | 7029649.816 |
| **WTB2** | .002 | 152146.241 | -312741.075 | 312741.079 |
| **WTB3** | -1.002e-5 | .000 | -2.801e-5 | 7.982e-6 |
| **SLH** | -144.187 | .000 | -144.187 | -144.187 |
| **KH** | 6.302e-5 | .004 | -.007 | .008 |
| **SLA** | -.001 | .063 | -.130 | .129 |
| **YIA** | 38.418 | .002 | 38.414 | 38.421 |
| **SLHR** | .002 | 3694937.999 | -7595053.829 | 7595053.833 |
| **YIHR** | 25.042 | .000 | 25.042 | 25.042 |
| **SLPV** | .062 | 845669.692 | -1738298.885 | 1738299.009 |
| **PVYI** | 21.226 | .000 | 21.226 | 21.226 |

**Table footnote: *** B0 - B3 = Ventilation coefficient values; WTB0-WTB3 = Weight coefficient values; SLH = Slope of Height, KH = The rate constant; SLA = Slope of Age; YIA = Y-intercept of age; SLHR = Slope of heart rate; YIHR = Y-intercept of heart rate, SLPV = Slope of peak oxygen uptake, PVYI = Y-intercept of peak oxygen uptake.

**Table S4:** Bootstrap confidence intervals for leave-one-out cross-validation (LOOCV) model coefficients predicting the oxygen cost of breathing (V̇O₂_VENT_)

| **Coefficient** | **Estimate** | **CI (95%)** |
| --- | --- | --- |
| (Intercept) | 5.74e+01 | [-5.36, 15.2] |
| V̇_E_ | -9.70e-04 | [-0.0616, 0.0734] |
| V̇_E_² | 1.77e-05 | [-0.000601, 0.000606] |
| V̇_E_³ | 1.00e-07 | [-1.44e-06, 1.68e-06] |
| Weight | -1.11e-01 | [-0.737, 0.153] |
| Weight² | 1.87e-03 | [-0.00226, 0.0106] |
| Weight³ | -1.00e-05 | [-5.01e-05, 1.07e-05] |
| exp(Height) | -1.44e+02 | [-9.41e-83, 4.34e-83] |
| Age | -5.36e-04 | [-0.0117, 0.00519] |
| V̇O₂peak | 6.22e-02 | [-0.0707, 0.0878] |
| HRMax | 1.88e-03 | [-0.0047, 0.0102] |

**Note.** Coefficients are reported with their 95% percentile-based bootstrap confidence intervals (CI) calculated from 500 resamples. V̇O₂_VENT_ = ventilatory oxygen cost; V̇_E_ = minute ventilation; VO₂peak = peak oxygen uptake; HRMax = maximal heart rate.
